# One‐day surgery is safe and effective in unicompartmental knee arthroplasty: A prospective comparative study at 1 year of follow‐up

**DOI:** 10.1002/ksa.12350

**Published:** 2024-07-11

**Authors:** Stefano Petrillo, Claudio Lacagnina, Michele Corbella, Matteo Marullo, Marco Bargagliotti, Riccardo Giorgino, Paolo Perazzo, Sergio Romagnoli

**Affiliations:** ^1^ Department of Joint Replacement IRCCS Ospedale Galeazzi San'Ambrogio Milan Italy; ^2^ Department of Orthopaedics Fondazione Istituto G. Giglio Cefalù Italy; ^3^ Residency Program in Orthopaedics and Traumatology University of Milan Milan Italy; ^4^ Intensive care Unit IRCCS Ospedale Galeazzi Sant'Ambrogio Milan Italy

**Keywords:** 1‐day surgery protocol, anteromedial or anterolateral knee osteoarthritis, early mobilisation, enhanced recovery after surgery, medial unicompartmental knee arthroplasty, pain management strategies

## Abstract

**Purpose:**

To compare the outcomes and complications of two perioperative protocols for the management of patients who underwent medial unicompartmental knee arthroplasty (UKA): 24 h (1‐day surgery [OS]) versus 72 h (enhanced recovery after surgery [ERAS]) of the length of hospital stay (LOS). In our hypothesis, the reduction of the LOS from 3 to 1 day did not influence the outcomes and complications.

**Methods:**

A total of 42 patients (21 in each group) with isolated anteromedial knee osteoarthritis and meeting specific criteria were prospectively included in the study. Clinical outcomes included Knee Society Score (KSS) and Forgotten joint score while pain evaluation was performed using a Visual Analogue Scale (VAS). Functional outcomes were assessed measuring the knee range of motion (ROM) while radiographic outcomes were evaluated measuring the amelioration of the varus deformity through the hip–knee–ankle angle (HKA).

**Results:**

Clinical and functional outcomes did not significantly differ between the two groups. Complications occurred in 9.5% of OS and 4.7% of ERAS group patients. Significant improvements in knee ROM, VAS pain, KSS and HKA angle were observed postsurgery, with no significant differences between groups except in KSS expectations and function trends.

**Conclusion:**

The OS protocol is safe and effective and LOS, in a well‐defined fast‐track protocol, did not significantly impact clinical and functional outcomes. OS may lead to reduced hospitalisation costs and potential reductions in complications associated with prolonged stays, benefiting both patients and healthcare facilities. However, further research with larger sample sizes and longer follow‐up periods is needed to confirm these findings. Early mobilisation and rehabilitation protocols are key components of successful patient recovery following UKA procedures.

**Level of Evidence:**

Level II.

**Abbreviations**
ERAS: enhanced recovery after surgery
FJS: Forgotten joint score
Hb: haemoglobin
HKA: hip–knee–ankle angle
IQR: interquartile range
KL: Kellgren–Lawrence
KSS: Knee Society Score
LIA: local infiltration analgesia
LOOS: Knee Injury and Osteoarthritis Outcome Score
LOS: length of hospital stay
OA: osteoarthritis
OS: 1‐day surgery
RIST: reduced instrumentation surgical technique
ROM: range of motion
TXA: tranexamic acid
UKA: unicompartmental knee arthroplasty
VAS: Visual Analogue Scale

## INTRODUCTION

The unicompartmental knee arthroplasty (UKA) is currently the gold standard for the management of patients with isolated anteromedial or anterolateral knee osteoarthritis (OA) [12, 23, 37] and has shown also excellent results in tibial or femoral condyle osteonecrosis. UKA surgery is performed through a smaller surgical incision, allowing for better bone preservation, improved range of motion (ROM), better proprioception, less postoperative pain and reduced surgical time, resulting in less blood loss, lower infection risk and postoperative complications [2, 25]. The morbidity and mortality rate after UKA is more than halved within the first 30 days following the surgery [27, 28] and hospitalisation time is reduced compared to total knee arthroplasty (TKA) [7]. Moreover, UKA has a lower likelihood of complications compared to TKA, with a higher rate of patient satisfaction at a short‐ and long‐term follow‐up. Nowadays there is a growing focus on reducing the length of hospital stay (LOS) and fast‐track protocols have been introduced and progressively implemented, especially in patients undergoing knee prosthetic surgery. The aim of such protocols was to reduce the complications related to prolonged hospitalisation and more recently, LOS for knee arthroplasty has significantly decreased, from an average of 5–7 days to about 3 days, thanks to the introduction and optimisation of specific protocols [18], such as the enhanced recovery after surgery (ERAS) pathway, already applied in various surgical specialties [42]. This protocol involves several stages of patient care, from preoperative preparation to minimally invasive surgery, anaesthesiologic procedures aimed at maximum protection from surgical stress, optimal pain control and early mobilisation. Considering this particular scenario, the use of UKAs facilitates early discharge procedures, since it was widely demonstrated that UKA efforts faster recovery, reduced morbidity, favourable short‐term adverse event and decreased postoperative pain compared to TKA. Several authors have reported good results of outpatients UKA, but no one of them had compared 1‐day surgery (OS) and ERAS after UKA.

The aim of our study was to compare the outcomes and complications in patients with UKA who have received two different fast‐track protocols: OS versus ERAS. Our hypothesis was that, in a fast‐track setting, a reduction of the LOS from 3 to 1 day does not influence the outcomes and complications of patients who underwent UKA.

## METHODS

**Figure 1:**
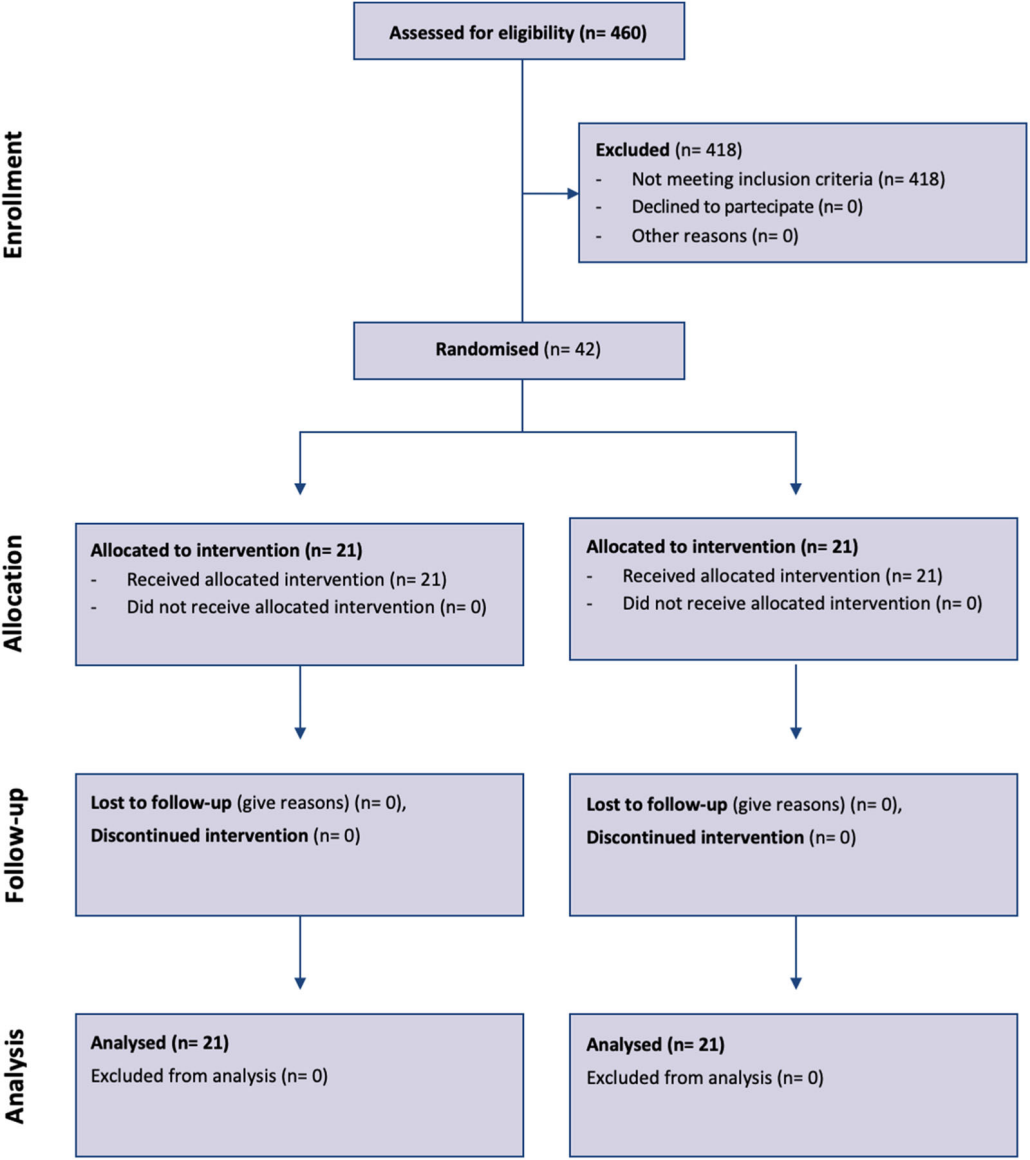
CONSORT diagram [39].

From March 2021 to May 2022, a total of 448 UKAs were performed in 460 patients in our Institution. Of these, 42 patients met inclusion/exclusion criteria (Table 1) and were prospectively included in the study after giving their written informed consent to participate (Figure 1).

**Table 1:** Inclusion and exclusion criteria.

Inclusion criteria	Exclusion criteria
Patients with residence in Lombardia Patients with residence in a neighbour region of Lombardia Age: female ≤ 70 years, male ≤ 75 years BMI ≤ 30 kg/m^2^ ASA Score I–III Support at home Physiotherapist available at home Patients motivated	Previous ipsilateral knee fracture Previous knee surgery Severe anaemia Drugs addiction Antidepressant therapy Bleeding disorders or NOAC Cardiovascular diseases Pregnancy Charlson Comorbidity Index ≤ 4

Abbreviations: ASA, American Society of Anaesthesiology; BMI, body mass index.

The patients were divided into two groups of 21 patients each, according to the LOS: Group A (24 h—OS) and Group B (72 h—ERAS).

### Operative protocol

All surgical procedures were performed with the patients under spinal anaesthesia in combination with adductor canal nerve block and without a tourniquet. A mini‐midvastus surgical approach and a reduced instrumentation surgical technique (RIST) [34] were used to implant a cemented medial UKA (Persona Partial Knee; Zimmer‐Biomet). The antibiotic prophylaxis (30 min before surgery) consisted of a single‐shot intravenous administration of Cefazolin with a dosage of 30 mg/kg. At the same time, 4 mg of Dexamethasone, along with 4 mg of Ondansetron and 40 mg of Omeprazole, were given for perioperative prevention of nausea and vomiting. Prior UKA implantation, a local infiltration analgesia (LIA) procedure was performed, using 0.2% Ropivacaine, 0.5 mg of Adrenaline, and 90 mg of Ketorolac, totalling 120 mL of the product [1]. Tranexamic acid (TXA) was used 15 min preoperatively (10–15 mg/kg) and another 1 g was injected into the knee at the end of the capsule suture. Moreover, additional intravenous therapy with TXA (10/15 mg/kg in a 250 mL saline solution at a rate of 50 mL/h for 5 h) is administered. At the end of the surgery, a drainage was positioned and removed 6 h after surgery and a compression bandage was used to reduce knee swelling and increase the effect of the LIA. Patients were then transferred to a recovery room and the knee was maintained at 30° of flexion in a cryotherapy machine to reduce postoperative bleeding for 2 h. Four hours after surgery, the patients started mobilisation and walking with two crutches bearing full weight on the operated leg. Then, a 0–90° continuous passive motion machine was positioned for another 2 h.

Perioperative pain management therapy was oral, consisting of Celecoxib 200 mg every 12 h and Paracetamol 1 g every 8 h. No opioids were admitted, and in case of severe uncontrolled pain (VAS > 7), Ketorolac 15 mg was administered (max. every 12 h).

### Outcomes

The Knee Society Score (KSS) [31] was used to evaluate clinical outcomes preoperatively, at 24 h, 2, 6, 12, weeks and 1 year after surgery. Pain evaluation was performed using a Visual Analogue Scale (VAS) [15] preoperatively, then at 6, 12 and 24 h, and 2, 6 and 12 weeks and 1 year after surgery. The Forgotten joint score (FJS) [11] was administered at the final follow‐up. Functional outcomes were assessed by measuring the knee ROM preoperatively and at 6, 12 and 24 h, and 2, 6 and 12 weeks and 1 year after surgery.

### Imaging

All patients underwent radiographic evaluation preoperatively, 1 h and 6 weeks after surgery. The following X‐ray views were obtained preoperatively and postoperatively: anteroposterior full‐length weight bearing of the lower limb; anteroposterior, lateral, axial patella at 45°° and Rosenberg projection of the knee. The radiographic data considered were preoperative grade of OA according to Kellgren–Lawrence classification; [21] preoperative and postoperative hip–knee–ankle angle.

### IRB approval

The present study was approved by the Ethics Committee board of San Raffaele University of Milan (IRB n. 55/INT/2021 of 10/03/2021).

### Statistical analysis

Statistical analysis was performed using GraphPad Prism v5.0 (Prism Software). The normal distribution of continuous variables was evaluated by Shapiro–Wilk test, and the data were reported in agreement with the test result: median and interquartile range in case of nonnormal distribution otherwise. Similarly, comparisons between groups in the univariate case were performed by Student's *t* test (normal distribution) or Mann–Whitney (nonnormal distribution). Multivariate analysis was performed using two‐way analysis of variance models, with Bonferroni post hoc test. Categorical variables were reported as absolute frequencies and any differences between groups were assessed by *χ* ^2^ test (or *χ* ^2^ test for trends, if appropriate). Values of *p* < 0.05 were considered statistically significant. An ‘a priori’ power analysis was carried out in order to assess the appropriateness of the sample of patients enroled. Considering that our objective was to compare outcomes with the KSS between the two groups of patients, at a minimum follow‐up of 12 + 2 weeks from medial UKA, applied an average test of a sample for noninferiority and considering a standard deviation of 9.7 points, as already shown in our previous study, and by a margin of five points, a total of 40 points were necessary for an *⍺* level test = 0.025 and a power = 90%.

## RESULTS

The two groups were homogeneous for all variables considered (Table 2). No patients were lost from the final follow‐up or had prolonged hospital stay. Complications occurred in 2 (9.5%) (grade 0 and 2 according to Goslings and Gouma criteria [14]) and 1 (4.7%) (grade 0 according to Goslings and Gouma criteria [14]) OS and ERAS group patients, respectively. Complications were not related to fast‐track protocols.

**Table 2:** Patients' characteristics.

	OS	ERAS	*p* Value
Age (years)	63.7 ± 6.7	64.4 ± 7.3	0.742
Sex	9 Females	8 Females	0.999
	12 Males	13 Males	
BMI (kg/m^2^)	25.9 ± 3.2	26.1 ± 2.5	0.823
KL			0.334
1	0	0	
2	6	2	
3	9	13	
4	5	5	
ASA Score			0.999
1	7	7	
2	13	14	
3	0	0	
Charlson Comorbidity Index (10 years mortality)	2.1 (86.6%)	2 (87.1%)	0.334
Surgery duration (min)	40.2 ± 7.0	42.2 ± 7.0	0.209
Drain (mL)	51.7 ± 52.3	57.1 ± 38.1	0.382
HB (mg/dL)	14.2 ± 0.9	14.1 ± 1.1	0.963

Abbreviations: ASA, American Society of Anaesthesiology; BMI, body mass index; ERAS, enhanced recovery after surgery; HB, haemoglobin; KL, Kellgren–Lawrence; OS, 1‐day surgery.

**Figure 2:**
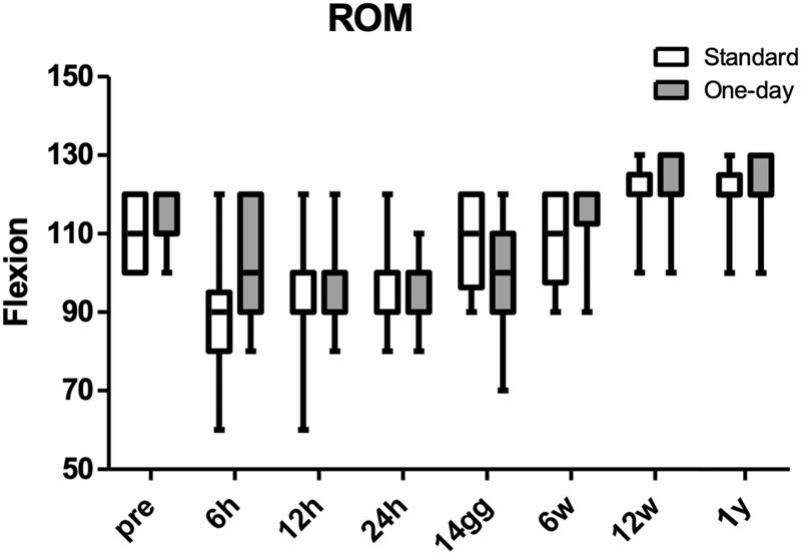
Preoperative and postoperative range of motion (ROM) values in the two groups.

**Figure 3:**
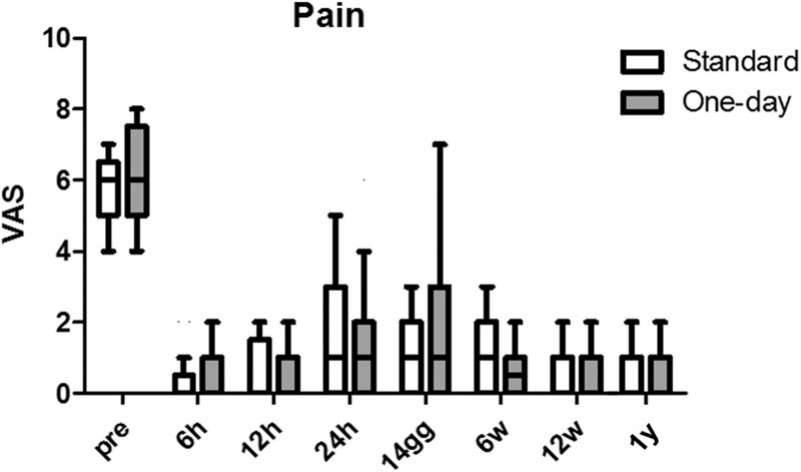
Preoperative and postoperative pain in the two groups, expressed on the Visual Analogue Scale (VAS).

The knee ROM and VAS significantly improved after surgery (*p* < 0.001), while no statistically significant differences were found comparing the two variables between the two groups (Figures 2 and 3).

**Figure 4:**
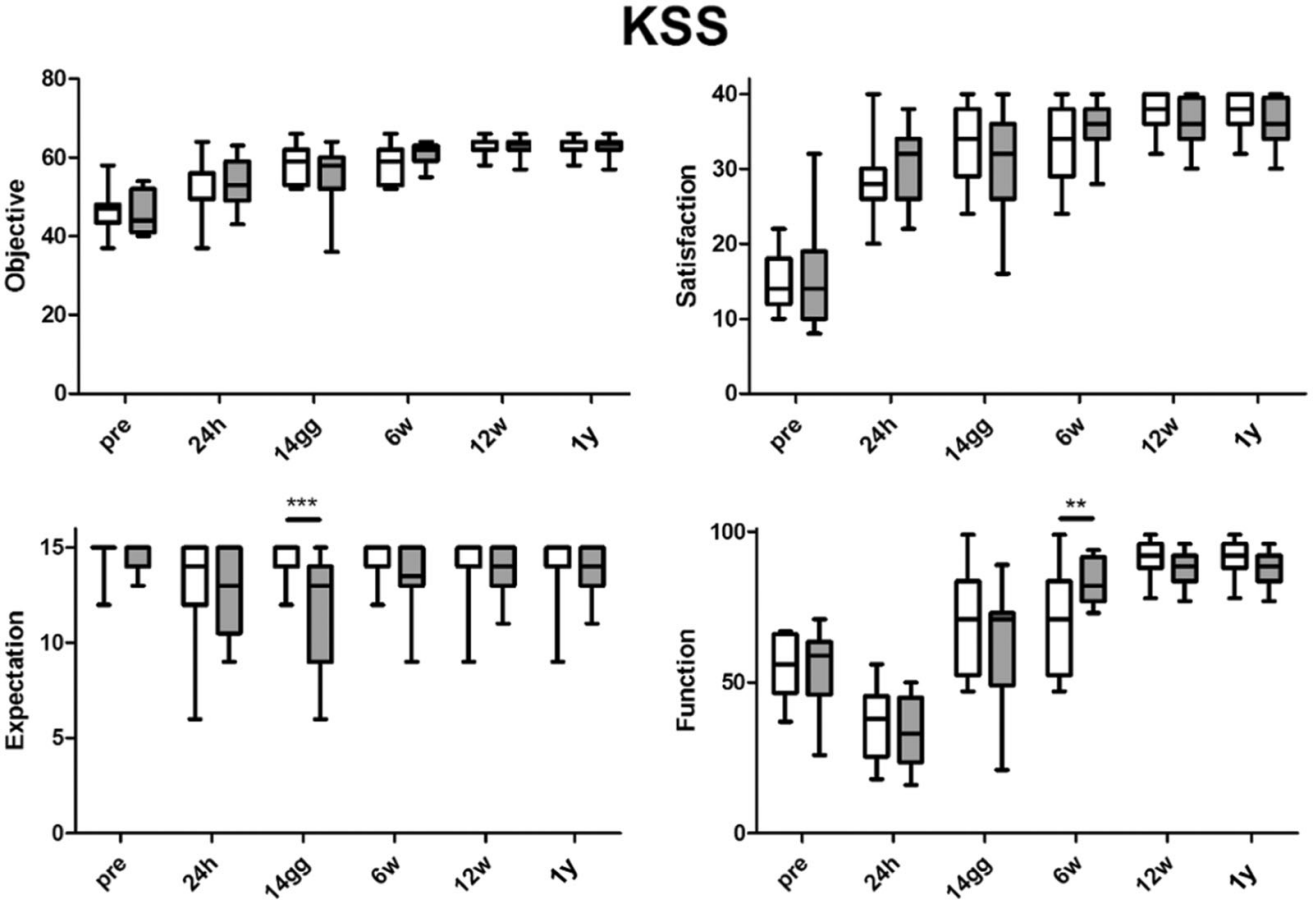
Preoperative and postoperative values of Knee Society Score (KSS) in the two groups. **p < 0.01; ***p < 0.001.

**Figure 5:**
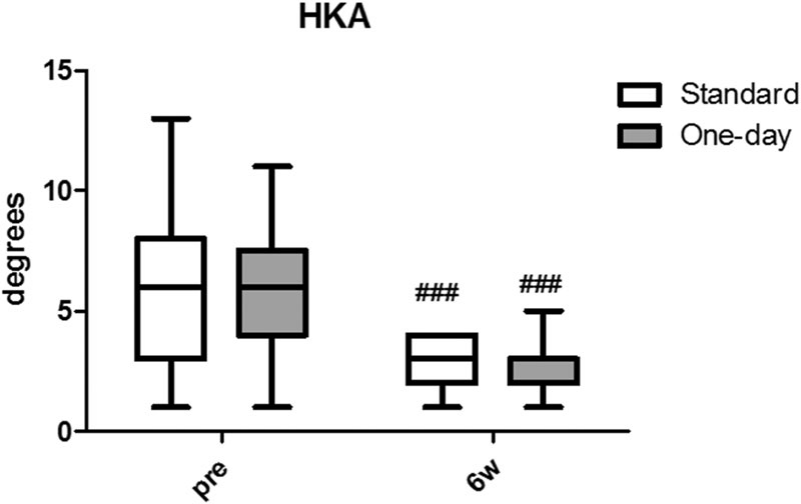
Preoperative and postoperative values of hip–knee–ankle angle (HKA) in the two groups. ^###^ *p* < 0.001 versus baseline within the same group.

**Figure 6:**
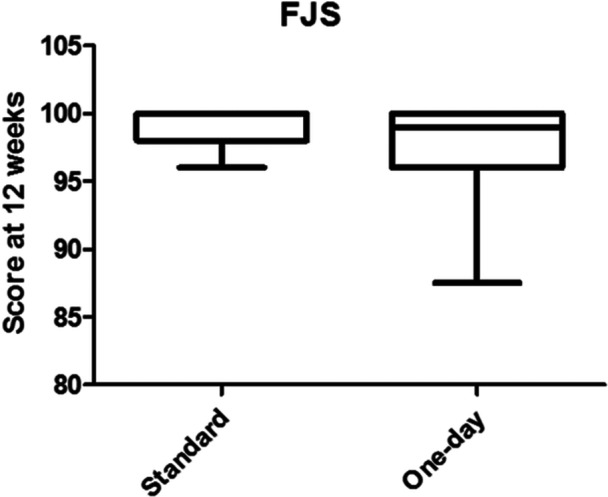
Values of Forgotten joint score (FJS) in the two groups at 1 year of follow‐up.

The objective and satisfaction sections of the KSS showed a significant improvement after surgery (*p* < 0.001), while no statistically significant differences were found between the two groups. Considering the KSS expectation and function section of KSS, significant differences were noticed in the trends between the two groups. Patients in the OS group had higher expectations, resulting in a significant difference at 14 days (*p* < 0.001). Regarding the function section of KSS, patients in the OS group demonstrated a faster respect to those in ERAS group (*p* < 0.01) (Figure 4). The varus deformity of the operated knee significantly decreases after surgery (*p* < 0.001), but no differences between the two groups were found (Figure 5). The comparison of FJS results was not significant between the two groups (Figure 6).

## DISCUSSION

The main finding of our study was that, in a fast‐track protocol setting, the reduction of the LOS from 3 to 1 day did not influence significantly the outcomes and complications rate after UKA. Our study investigated the outcomes of UKA surgery at 1‐year follow‐up comparing two groups of 21 patients each: OS (24‐h hospital stay) versus ERAS (72‐h stay). The results showed that both groups had comparable rates of complications and hospital readmissions, affirming the safety and effectiveness of both protocols. Moreover, clinical scores (KSS‐o, KSS‐s) did not exhibit significant differences between the two groups and improved significantly over the time, suggesting that the LOS following minimally invasive medial UKA did not impact clinical outcomes. However, in the expectations section of the KSS, reflecting patients' expectations, lower scores were observed in the study group, especially 14 days after surgery, suggesting that sometimes patients mistakenly the shorter hospital stay with quicker recovery. On the other hand, when considering the function section of the KSS, patients in the OS group had better results, demonstrating that a shorter LOS is safe and effective. Pain levels (VAS) were similar in both groups, indicating that pain control was influenced more by surgical techniques and anaesthesia than hospital stay duration. ROM improved significantly over time, with no significant differences between the groups, suggesting that the recovery of knee ROM was not affected by early discharge. Functional scores (FJS) at 1‐year postsurgery did not differ significantly between the groups. The preoperative and postoperative radiographic analysis confirmed the correct execution of the surgical procedures in both groups, with no significant differences in radiographic data.

Most previous studies in this area primarily examined outpatient or day‐off hospital stay pathways, which are currently prohibited by the Italian national healthcare system, making it challenging to compare their results [3, 8, 10, 13, 33, 36]. Two studies similar to the current one were conducted by Kort et al. [22] and Hoorntje et al. [16]. Kort et al. divided 40 patients into two groups, one having outpatient stays and the other averaging 2.6 days in the hospital. They found outpatient pathways safe and effective if patients were carefully selected, although they reported a few delayed discharges due to postoperative pain control and patient concerns about returning home. They also noted a hospital readmission case due to a knee extension deficit. In contrast, the current study experienced no delayed discharges but had one hospital readmission due to an early infection [22]. Hoorntje et al. also considered outpatient stays safe and emphasised patient selection [16]. They used various scales to evaluate patients, such as the Numeric Rating Scale [4], Hospital Anxiety and Depression Scale [29], Oxford Knee Score [40] and Knee Injury and Osteoarthritis Outcome Score [5]. Their study had one case of hospital readmission due to surgical wound dehiscence, but they found no significant differences between the two groups. The effectiveness of an optimised protocol in reducing hospitalisation time and perioperative/postoperative complications undoubtedly depends on a collaborative and resource‐adaptive approach led by a multidisciplinary team [6].

The importance of controlling postoperative pain, nausea and bleeding for early discharges was highlighted and a multimodal opioid‐sparing analgesic protocol, along with intraoperative LIA, was considered essential [42]. Our protocol avoided the undesirable effects of opioids and used the adductor canal nerve block for effective pain control. Regarding LIA, existing literature strongly supports the use of LIA, which helps reduce postoperative pain intensity, opioid consumption and the overall duration of hospital stays [1, 42, 43]. When combined with the adductor canal nerve block, LIA demonstrates even better pain control within 18 h after surgery, leading to an improved postoperative ROM and early mobilisation [30, 44]. Moreover, Sawhney et al. [38] demonstrated that combining LIA with the adductor canal nerve block has shown better control of postoperative pain than using LIA or the adductor canal nerve block alone. Comparing our study's findings with a previous research by Munk et al. [32], it is evident that our approach to postoperative pain management was more effective. In Munk's study, patients undergoing medial UKA had an average hospital stay of 1.1 days, and many reported moderate to severe pain levels, especially on the first postoperative day, leading to the use of opioid medications [32]. In contrast, our study's patients had notably lower pain levels, with average VAS scores of 2.09 ± 1.97 in group A and 1.18 ± 1.66 in group B 24 h postsurgery. This improved pain control can be attributed to the study's anaesthetic protocol, which incorporated LIA in conjunction with the adductor canal nerve block without using opioids. Additionally, the effectiveness of the adductor canal nerve block over the femoral nerve block is widely established, as it maintained quadriceps femoris strength while providing comparable analgesia, thereby reducing the risk of falls and enabling early patient mobilisation [17, 20, 41].

TXA played a significant role in our study's anaesthetic protocol by preventing excessive perioperative blood loss, consequently reducing the need for postoperative transfusions during hospitalisation [9, 19, 26, 44]. Unlike some cases in which patients require transfusions, none of the patients in the study required postoperative transfusions, further contributing to the feasibility of early discharge and the prevention of hospital readmissions. The analysis of the study's results revealed no statistically significant differences in postoperative haemoglobin (Hb) values between the two groups and the average decrease in Hb values 24 h after surgery was negligible in both groups.

Considering our surgical procedure, we did not employ a tourniquet during surgery, a practice supported by existing literature that suggests its absence leads to faster postoperative recovery, improved joint ROM, better pain control and reduced analgesic use [34]. However, it remains uncertain whether its limited use provides additional meaningful benefits in reducing pain and early functional restoration after the ERAS protocol [24]. Our study employed a ‘RIST’, [35] which gradually corrects axial deformities while balancing ligaments, thereby reducing surgical time and intraoperative bleeding, often attributed to intramedullary guides and pins used for guide fixation [35]. The rehabilitation protocol prioritised early mobilisation, emphasising its importance in reducing vascular‐related complications like deep vein thrombosis and pulmonary embolism. Moreover, early mobilisation contributed to re‐establishing proper joint proprioception and gait kinematics from the beginning, positively impacting patients' psychological well‐being.

The study had several strengths, including its prospective design with a control group, standardised anaesthetic protocol, surgical technique and prosthetic type. Clinical, functional and radiological evaluations were conducted by a physician not directly involved in the surgeries. The study achieved a 100% follow‐up rate, with no patients experiencing surgery‐related severe complications or dropping out. Finally, conducted at a high‐volume national reference centre for knee prosthetic surgery, the study adhered to high safety standards.

However, the study had some limitations. First, the small sample size of only 42 patients may limit the generalisability of the results to a broader population. Additionally, the lack of randomisation could introduce selection bias, influencing the outcomes. Second, the 1‐year follow‐up duration might not be sufficient to detect long‐term differences between the two groups. Studies with longer follow‐up periods could have provided more comprehensive data. Furthermore, the effect of varying surgical experience amongst participating surgeons was not controlled for, which could impact postoperative results and complication rates. Moreover, some assessments, such as patient satisfaction, were based on subjective scales that could be influenced by psychological factors or individual perceptions of recovery. Finaltly, the results may not be universally applicable, as they were specific to a high‐volume national reference centre experienced in minimally invasive knee prosthetic surgery, which might not be replicable in other hospital settings.

In daily clinical practice, our findings emphasise that adopting a 24‐h hospital stay protocol (OS) following medial UKA surgery can provide comparable clinical and functional outcomes to traditional 72‐h stays (ERAS), while potentially reducing healthcare costs and hospital resource utilisation.

## CONCLUSION

In conclusion, the study suggests that OS may represent the next step in the natural evolution of ERAS protocols for medial UKA procedures. Both patient groups significantly improved KSS, pain control and ROM. The study affirms that OS is safe and effective, yielding clinical and functional results that align with ERAS protocols and demonstrating similar rates of complications and hospital readmissions. Further prospective comparative studies involving larger patient samples and longer follow‐up periods are required to draw definitive conclusions.

## AUTHOR CONTRIBUTIONS

**Stefano Petrillo**: Conceptualisation; methodology; investigation; writing—original draft preparation; writing—review and editing. **Claudio Lacagnina**: Conceptualisation; methodology; investigation; writing—original draft preparation. **Michele Corbella**: Investigation. **Matteo Marullo**: Investigation. **Marco Bargagliotti**: Investigation. **Riccardo Giorgino**: Methodology; writing—original draft preparation; writing—review and editing. **Paolo Perazzo**: Supervision. **Sergio Romagnoli**: Conceptualisation; investigation; supervision. All authors have read and agreed to the published version of the manuscript.

## CONFLICT OF INTEREST STATEMENT

The authors declare no conflict of interest.

## ETHICS STATEMENT

This protocol will be conducted in accordance with ethical principles originating from the Declaration of Helsinki, in compliance with Good Clinical Practice and applicable regulatory provisions. The Ethics Committee expresses a favourable opinion and requests that the information indicated by the Bioethicist be included in the Informed Consent.

## Data Availability

The original contributions presented in the study are included in the article/Supporting Information, further inquiries can be directed to the corresponding author.
